# Does zero-profile anchored cage accompanied by a higher postoperative subsidence compared with cage-plate construct? A meta-analysis

**DOI:** 10.1186/s13018-020-01711-9

**Published:** 2020-05-24

**Authors:** Yingjie Lu, Yuepeng Fang, Xu Shen, Dongdong Lu, Liyu Zhou, Minfeng Gan, Xuesong Zhu

**Affiliations:** 1 Department of Orthopedic Surgery, The First Affiliated Hospital of Soochow University, 899 Pinghai Road, Suzhou, 215000 China; 2 Department of Orthopedic Surgery, Suzhou Dushuhu Public Hospital (Dushuhu Public Hospital Affiliated to Soochow University), Suzhou, China

**Keywords:** Anterior fusion, Cervical degenerative disease, Zero-profile device, Subsidence, Meta-analysis

## Abstract

**Background:**

The zero-profile anchored cage (ZP) has been widely used for its lower occurrence of dysphagia. However, it is still controversial whether it has the same stability as the cage-plate construct (CP) and increases the incidence of postoperative subsidence. We compared the rate of subsidence after anterior cervical discectomy and fusion (ACDF) with ZP and CP to determine whether the zero-profile device had a higher subsidence rate.

**Methods:**

We performed a meta-analysis of studies that compared the subsidence rates of ZP and CP. An extensive and systematic search covered the PubMed and Embase databases according to the PRISMA guidelines and identified ten articles that satisfied our inclusion criteria. Relevant clinical and radiological data were extracted and analyzed by the RevMan 5.3 software.

**Results:**

Ten trials involving 626 patients were included in this meta-analysis. The incidence of postoperative subsidence in the ZP group was significantly higher than that in the CP group [15.1% (89/588) versus 8.8% (51/581), OR = 1.97 (1.34, 2.89), *P* = 0.0005]. In the subgroup analysis, we found that the definition of subsidence did not affect the higher subsidence rate in the ZP group. Considering the quantity of operative segments, there was no significant difference in the incidence of subsidence between the two groups after single-level fusion (OR 1.43, 95% CI 0.61–3.37, *P* = 0.41). However, the subsidence rate of the ZP group was significantly higher than that of the CP group (OR 2.61, 95% CI 1.55–4.40, *P* = 0.0003) after multilevel (≥ 2-level) procedures. There were no significant differences in intraoperative blood loss, JOA score, NDI score, fusion rate, or cervical alignment in the final follow-up between the two groups. In addition, the CP group had a longer operation time and a higher incidence of dysphagia than the ZP group at each follow-up time.

**Conclusion:**

Based on the limited evidence, we suggest that ZP has a higher risk of postoperative subsidence than CP, although with elevated swallowing discomfort. A high-quality, multicenter randomized controlled trial is required to validate our results in the future.

## Introduction

Anterior cervical discectomy and fusion (ACDF) is the standard surgical procedure for cervical degenerative disk disease (CDDD) [1]. The use of the anterior plate can increase the stability of the cervical spine and promote early intervertebral fusion after surgery, and it is widely used in clinical practice [2–4]. However, studies over the past decade have shown that the use of anterior plates can increase the incidence of postoperative dysphagia, and even cause long-term unremitting swallowing discomfort [5–8]. To solve this complication, many researchers have adopted a variety of new self-locking zero-profile anchored cages (ZPs), such as the Zero-P, MC+, and ROI-C [9–13]. These types of devices can reduce the compression of prevertebral soft tissue, and have similar stability and clinical efficacy as traditional cage-plate constructs [14–16]. Additionally, the new devices are easy to place and can reduce the operation time and blood loss significantly [10, 13], so their clinical application has gradually increased in recent years.

However, some studies have found that these ZPs are prone to subsidence because they do not have a strong fixation ability to maintain the height of the intervertebral space [9, 11]. This may lead to narrowing of the foramen, reducing of cervical lordosis, and neurological symptoms, affecting the long-term efficacy of patients [17–20]. At present, there is no consistent conclusion about whether the ZP has a higher subsidence rate than the traditional cage-plate construct (CP) in the literature.

Therefore, we reviewed the literature and conducted this meta-analysis to compare the postoperative subsidence and other clinical outcomes of ACDF with the ZP and CP; we aimed to determine whether the ZP has a higher postoperative subsidence rate and similar clinical efficacy as the CP.

## Materials and methods

### Search strategy

Our research complied with the guidelines of Preferred Reporting Items for Systematic Reviews and Meta-Analysis (PRISMA) [21, 22]. The electronic PubMed and Embase databases were searched from January 01, 2010 until April 30, 2019. The search was conducted with the following search strategy: “zero-profile[Title]” OR “Zero-P[Title]” OR “self-locking[Title]” OR “anchored spacer[Title]” OR “stand-alone cage[Title]” AND “cervical[Title]”. According to the selection methods and the inclusion criteria, the relevant articles and their references were reviewed.

### Selection criteria

All systematic reviews and meta-analysis on clinical controlled trials for CDDD were reviewed. The inclusion criteria for studies in this research were as follows: (1) patients had a failure of at least 6 months of non-operative treatments for CDDD; (2) the studies included a comparison of patients who accepted a ZP with those who received a CP; (3) the studies analyzed and compared the radiological outcomes of subsidence; (4) the studies involved an evaluation of an index of clinical effects: Japanese Orthopaedic Association (JOA) score, Neck Disability Index (NDI) score, operative time, intraoperative blood loss, fusion rate, cervical alignment, and complications; and (5) there was a follow-up period of more than 12 months.

We excluded studies that were not in line with clinical controlled studies, such as case reports, meta-analysis, conference abstracts, reviews, commentaries, and letters to the editor. In addition, we only included English language articles in this study.

### Data extraction

Articles from the literature were thoroughly searched and reviewed from by two reviewers (YJL and YPF) individually and repeatedly. Any discord was resolved through consultation with a third reviewer (XSZ). The data extracted from the article text, tables, and graphs of the qualified studies. The primary radiological data included subsidence, fusion rate, and cervical alignment, and the primary clinical data included JOA, NDI, operative time, blood loss, and the rate of dysphagia. The other basic data included study design, sample size, characteristics of patients, follow-up duration, and type of implants. If partial information from a study was missing, the corresponding author was contacted for the missing data.

### Statistical analysis

Data analysis was completed by using the Review Manager Software (RevMan 5.3, the Cochrane Collaboration). The continuous variables (operative time, blood loss, JOA, NDI, and cervical alignment) were evaluated by weighted mean difference (WMD), while dichotomous variables (incidence of subsidence, fusion rate, and dysphagia) were evaluated by odds ratio (OR). Considering the different definitions of subsidence (≥ 2 mm or ≥ 3 mm) and quantity of operative segments (1-level or ≥ 2-level) used in the different studies, we performed corresponding subgroup analysis upon incidence of subsidence. The *I*^2^ statistic was used to reflect the degree of heterogeneity. An *I*^2^ statistic > 50% identified obvious heterogeneity, and random-effects models were performed in these instances. If the *I*^2^ statistic was ≤ 50% (low heterogeneity), fixed-effects models were used. The study also used a funnel plot to evaluate publication bias for subsidence. A *P* value of 0.05 or less was considered statistically significant.

## Results

### Literature search and study characteristics

**Fig. 1:**
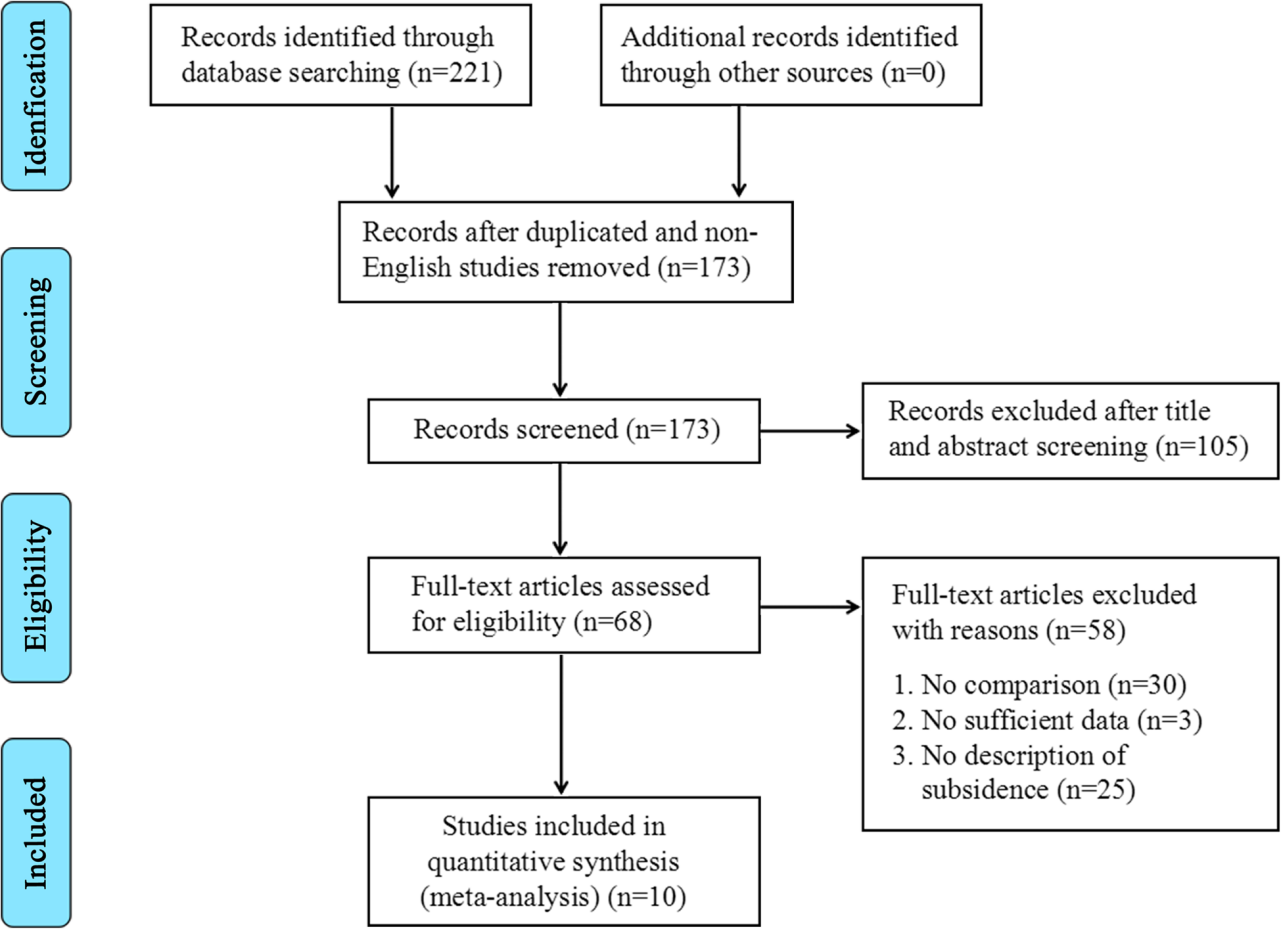
The graph shows the flow diagram of search strategy according to guidelines of PRISMA

A total of 221 related records were obtained by searching the databases above. After removing 26 duplicated studies and 22 non-English language articles, 173 studies remained for screening and 163 records were excluded according to the selection criteria. As a result, ten controlled trials [2, 9–13, 23–26] were included in this meta-analysis. The literature search procedure is shown in Fig. 1. All clinical trials came from different research centers. The study data and baseline characteristics of both treatment groups are presented in Table 1.

**Table 1:** Summary of study characteristics and demographics

Study	Type	Time	Study period	Country	Surgical levels (ZP/CP)	Patients (ZP/CP)	Gender (male/female)		Mean age		Follow-up (months)	Design of zero-profile device
							ZP	CP	ZP	CP		
Nemoto [12]	RCT	2015	2010–2012	Japan	One, 24/22	46 (24/22)	21/3	21/1	40.9 ± 7.2	41.6 ± 7.0	24	PREVAIL
Shin [24]	Retro	2014	2008–2013	Korea	One, 20/20	40 (20/20)	7/13	13/7	50.0 ± 12.0	44.3 ± 9.7	13.5	Zero-P
Lee [2]	Retro	2015	2005–2011, 2012–2013	Korea	One, 23/18	41 (23/18)	11/12	11/7	57.26 ± 13.28	52.89 ± 7.71	19.9	Zero-P
Shi [9]	Retro	2015	2010–2011	China	Three, 18/20	38 (18/20)	11/7	12/8	56.2 ± 64.8	56.7 ± 63.9	30.3	Zero-P
Chen [11]	Retro	2016	2010–2012	China	Three, 18/20	71 (33/38)	18/15	21/17	49.3 ± 3.7	48.8 ± 3.9	30.8	Zero-P
Li [13]	Retro	2017	2009–2013	China	One, 32/34; two, 21/23; three, 11/10; four, 4/3	138 (68/70)	41/27	45/25	50.6 ± 7.5	51.3 ± 7.9	24	Fidji
Yun [25]	Retro	2017	2006–2015	Korea	Two, 31/32	63 (31/32)	22/9	29/3	53.29 ± 7.55	54.18 ± 9.87	13.2	Zero-P
Zhou [10]	Retro	2018	2010–2013	China	One, 20/21; two, 18/14; three, 13/12	98 (51/47)	23/28	22/25	62.3 ± 6.7	64.4 ± 3.2	36	ROI-C
Lu [23]	Retro	2018	2011–2015	China	Two, 22/24	46 (22/24)	13/9	15/9	56.6 ± 6.4	58.6 ± 7.2	24	ROI-C
Zhu [26]	Retro	2019	2013–2014	China	Three, 30/32	62 (30/32)	12/18	18/14	56.6 ± 12.6	55.3 ± 11.3	36	MC+

*ZP* zero-profile group, *CP* cage-plate group, *RCT* randomized controlled study, *Retro* retrospective study

### Patient demographics

The 10 studies enrolled a total of 626 patients (315 in the ZP group and 311 in the CP group), which included 380 males and 236 females. The ZPs used in the studies included the Zero-P (Synthes GmbH, Oberdorf, Switzerland), ROI-C, ROI-MC+ (LDR, Troyes, France), PREVAIL (Medronic Sofamor Danek, Memphis, TN, USA), and the Fidji cervical cage (Abbott Spine, Bordeaux, France). The control group was treated with an anterior plating system and bone graft materials. The mean age, sex, follow-up durations, surgical levels, and other patient information from each study are listed in Table 1.

### Operative data

**Fig. 2:**
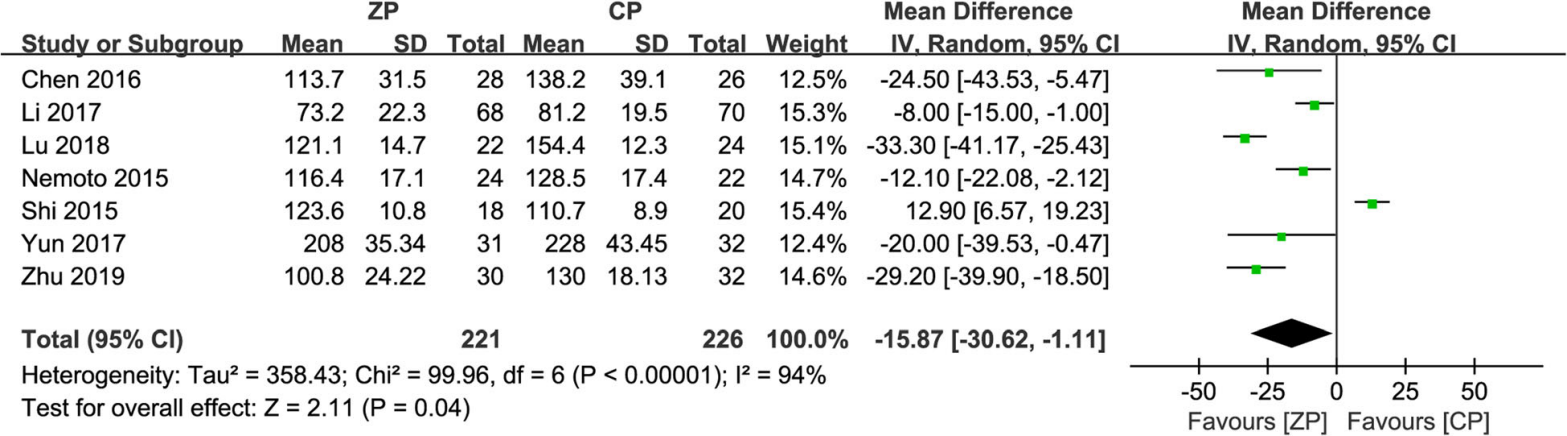
The forest plot shows operation time of anterior cervical discectomy and fusion by using zero-profile anchored cage versus cage-plate construct

Seven studies consisting of 447 patients (ZP group, 221; CP group, 226) noted the operation time [9, 11–13, 23, 25, 26]. The mean operation time was greater for the CP group in six studies, and after meta-analysis, the operation time was significantly greater in the CP group than in the ZP group (WMD − 15.87, 95% CI − 30.62 to − 1.11, *P* = 0.04) (Fig. 2).

**Fig. 3:**
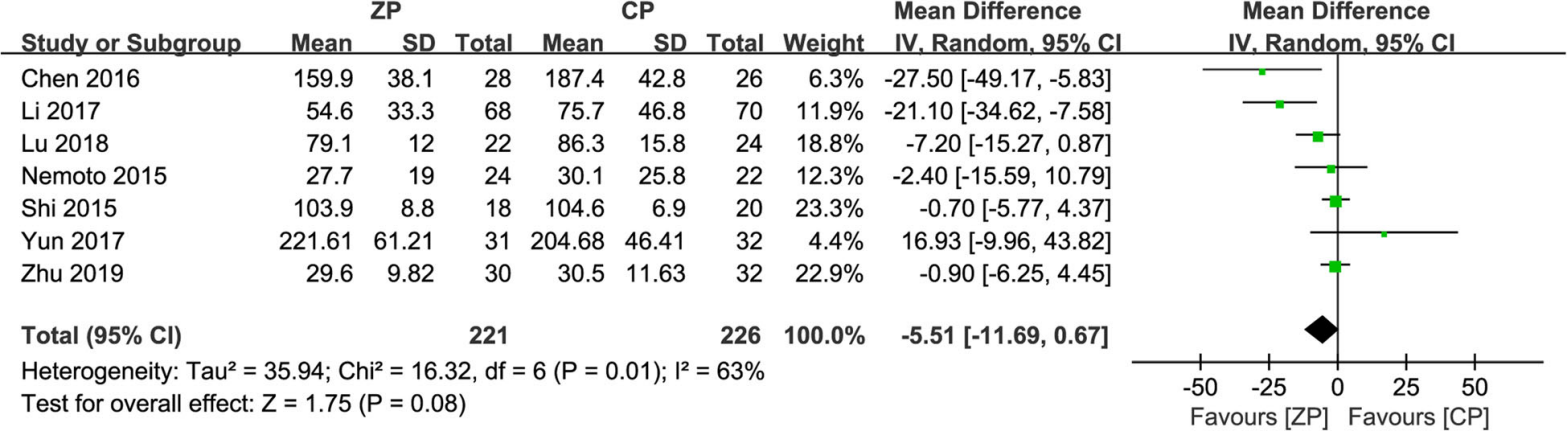
The forest plot shows intraoperative blood loss of anterior cervical discectomy and fusion by using zero-profile anchored cage versus cage-plate construct

For the outcome regarding intraoperative blood loss, seven studies including 447 patients (ZP group, 221; CP group, 226) reported this variable [9, 11–13, 23, 25, 26]. The ZP group was noted to have lower blood loss in six studies. Overall, the CP group had a comparable amount of blood loss versus the ZP group (WMD − 5.51, 95% CI − 11.69 to 0.67, *P* = 0.08) (Fig. 3).

### Clinical evaluation

**Fig. 4:**
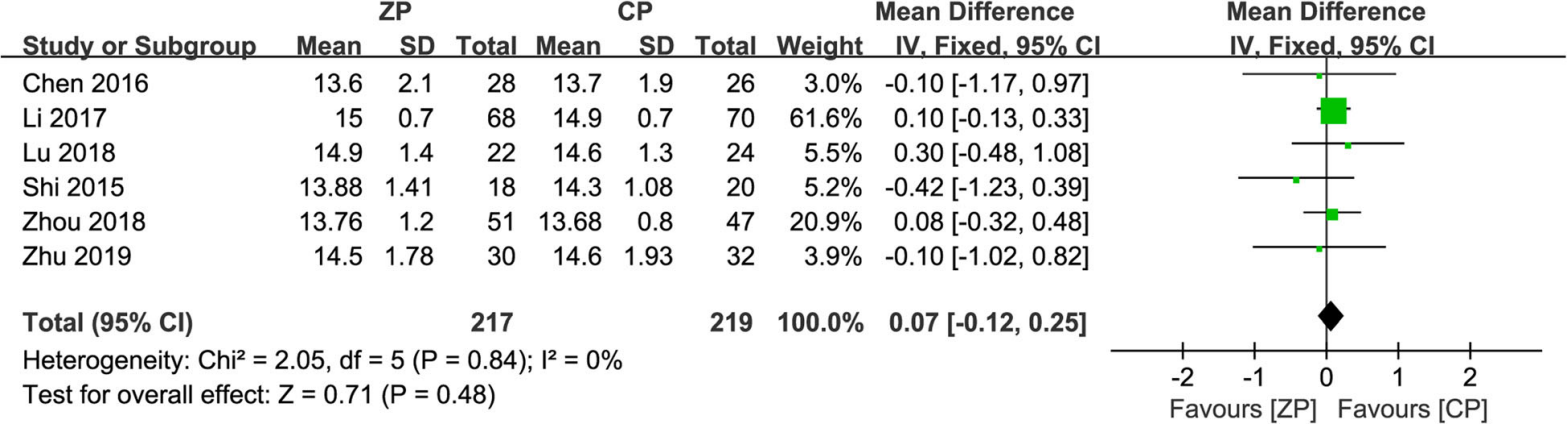
The forest plot shows JOA score of anterior cervical discectomy and fusion by using zero-profile anchored cage versus cage-plate construct at final follow-up

**Fig. 5:**
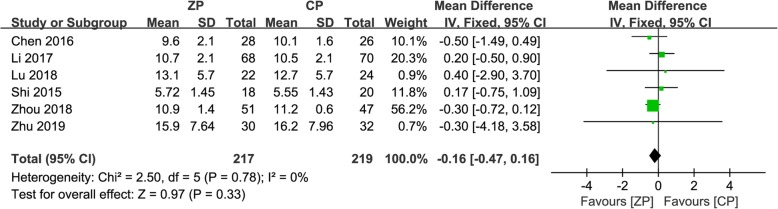
The forest plot shows NDI score of anterior cervical discectomy and fusion by using zero-profile anchored cage versus cage-plate construct at final follow-up

Data regarding JOA and NDI scores postoperatively were documented in six studies consisting of 436 patients (ZP group, 217; CP group, 219) [9–11, 13, 23, 26]. The mean difference in JOA scores at the final follow-up between the ZP and CP groups was not significant (WMD 0.07, 95% CI − 0.12 to 0.25, *P* = 0.48). In addition, pooled NDI score data at the final follow-up did not reveal a significant difference between the two groups (WMD − 0.16, 95% CI − 0.47 to 0.16, *P* = 0.33). Figures 4 and 5 describe the above information in forest plots.

### Radiological assessment

**Fig. 6:**
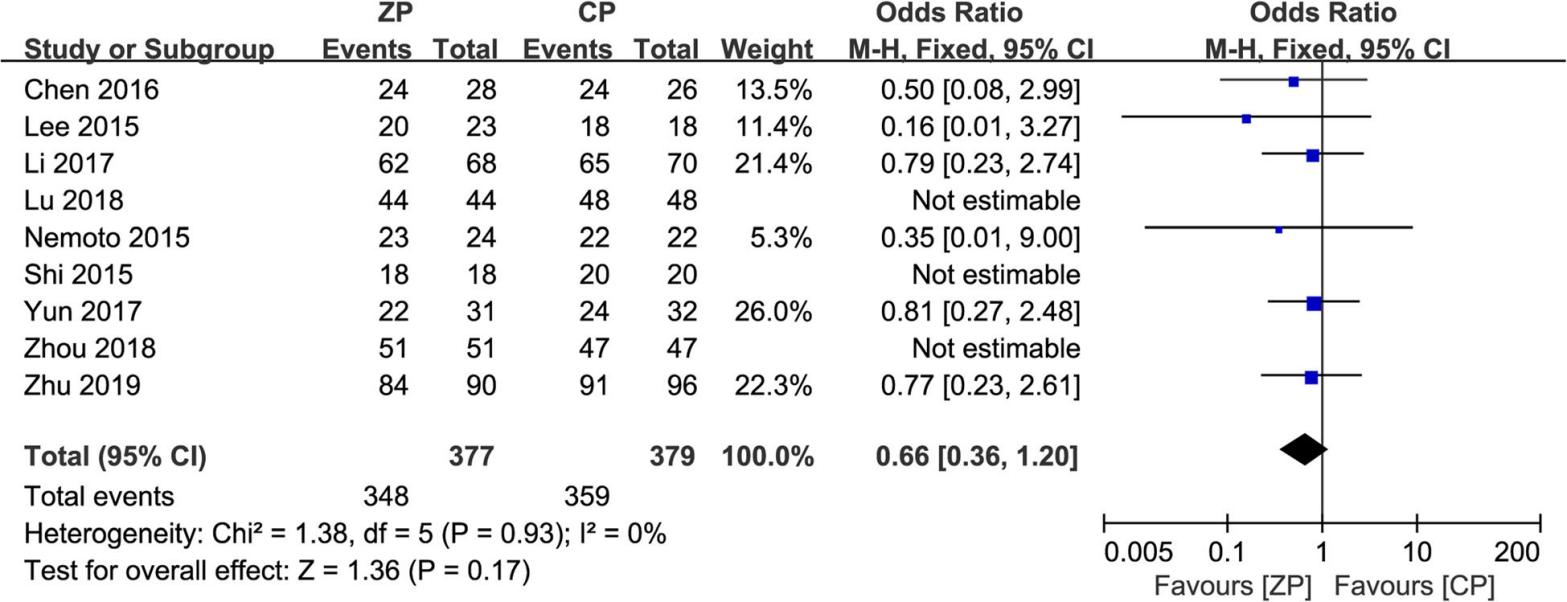
The forest plot shows the fusion rate of anterior cervical discectomy and fusion by using zero-profile anchored cage versus cage-plate construct at final follow-up

The results of radiographic fusion were described in nine studies [2, 9–13, 23, 25, 26], with fusion rates varying from 71 to 100%. Successful bone union was achieved in 348/377 patients (92.3%) in the ZP group, and 359/379 patients (94.7%) in the CP group. The forest plot analysis showed no significant difference between the two groups (OR 0.66, 95% CI 0.36 to 1.20, *P* = 0.17) (Fig. 6).

**Fig. 7:**
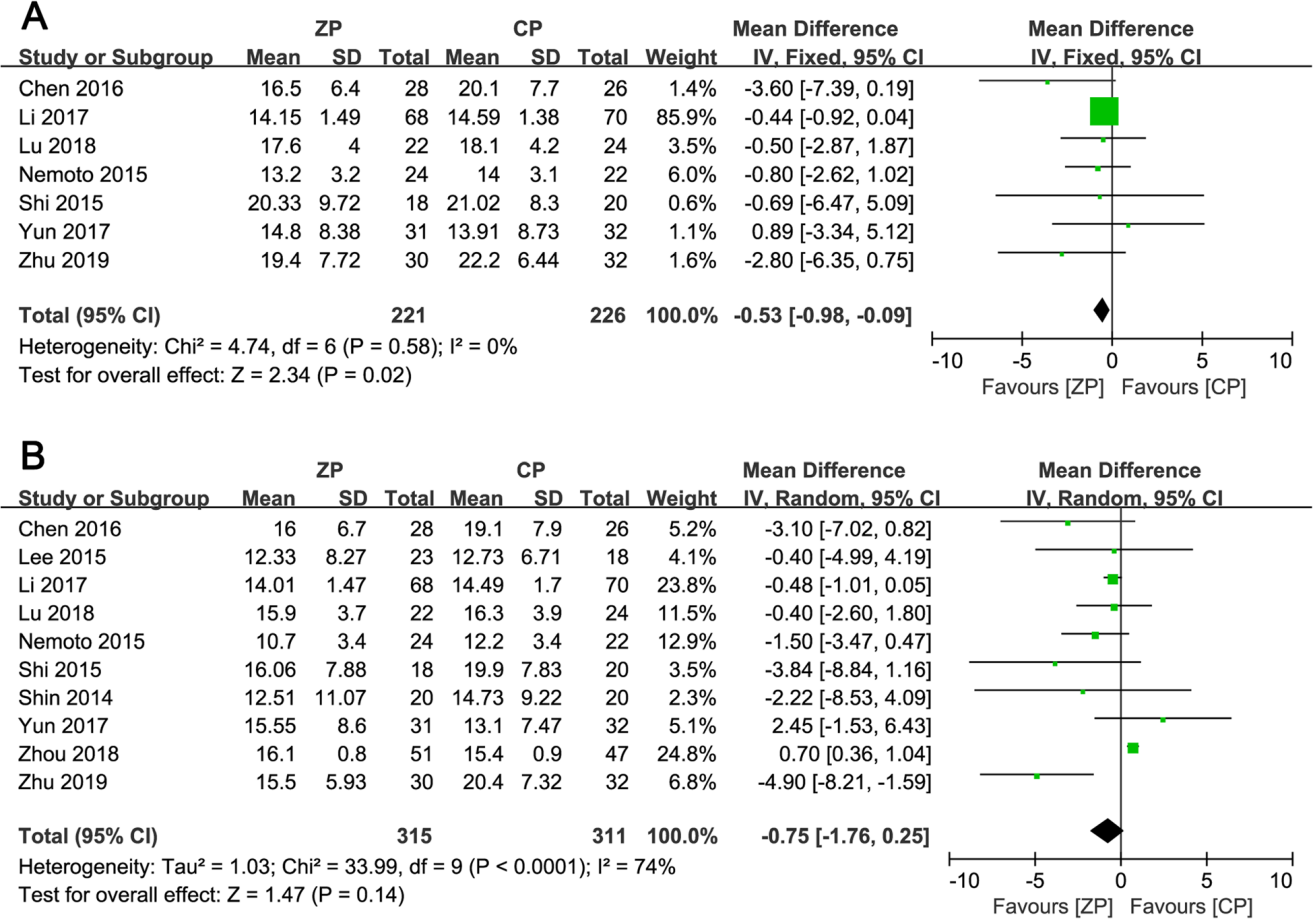
**a**, **b** The forest plots show cervical alignment of anterior cervical discectomy and fusion by using zero-profile anchored cage versus cage-plate construct at 3 months postoperatively (**a**) and final follow-up (**b**)

There was a significant difference regarding cervical alignment 3 months postoperatively between the ZP and CP groups (WMD − 0.53, 95% CI − 0.98 to − 0.09, *P* = 0.02). Nevertheless, the mean difference in cervical alignment between the two groups at the final follow-up was not significant (WMD − 0.75, 95% CI − 1.76 to 0.25, *P* = 0.14). The corresponding forest plot analysis is shown in Fig. 7.

### Dysphagia

**Fig. 8:**
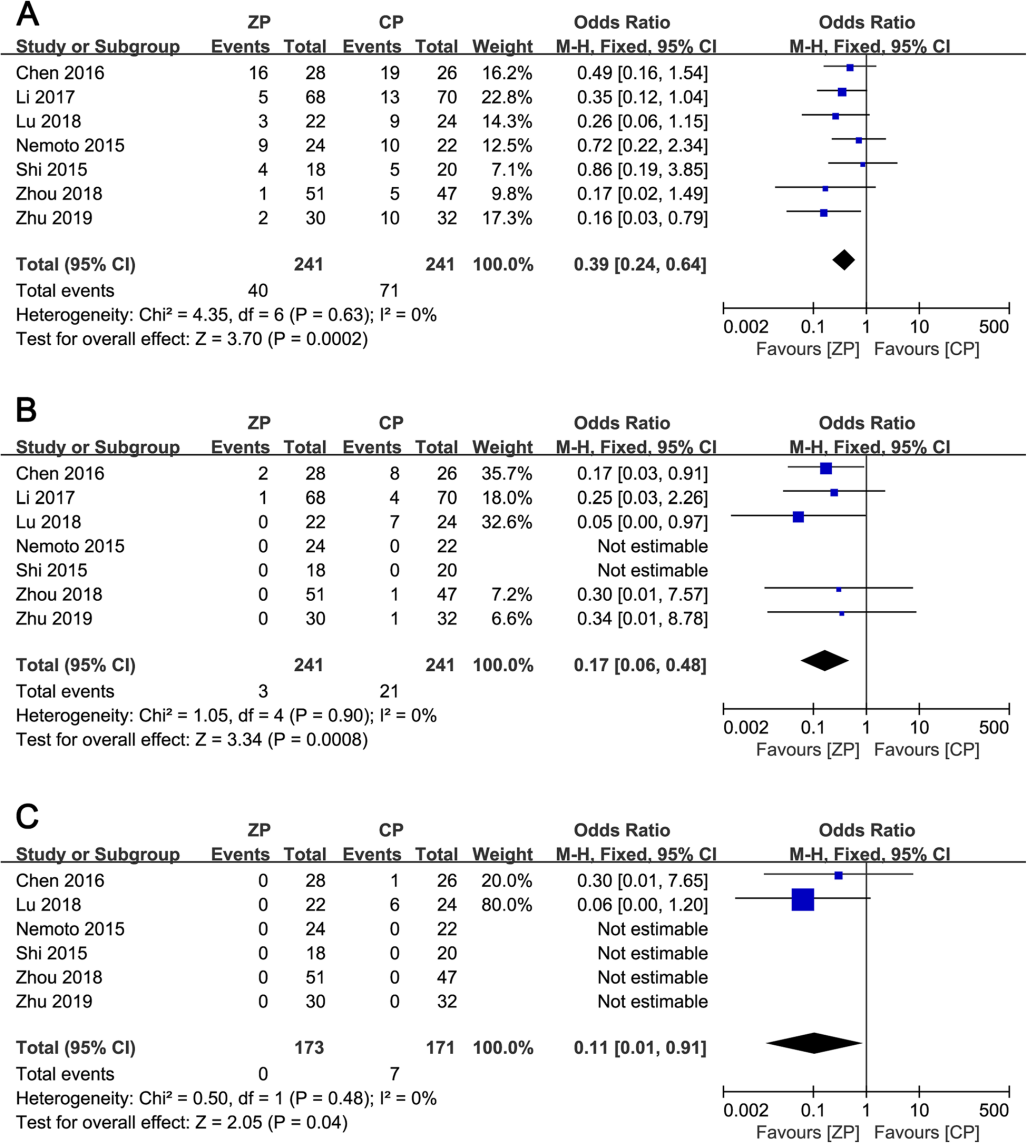
**a**–**c** The forest plots show the dysphagia of anterior cervical discectomy and fusion by using zero-profile anchored cage versus cage-plate construct at early postoperatively (**a**), 3 months postoperatively (**b**), and the final follow-up (**c**)

The incidence of dysphagia was reported in seven studies [9–13, 23, 26], with results of 2.0–57.1% in the ZP group and 10.6–73.1% in the CP group at the early period, respectively. In the early postoperative period (< 1 month), the rate of dysphagia was noted to be lower in the ZP group (OR 0.39, 95% CI 0.24 to 0.64, *P* = 0.0002). In addition, the ZP group also had a lower incidence of dysphagia at 3 months postoperatively (OR 0.17, 95% CI 0.06 to 0.48, *P* = 0.0008) and at the final follow-up (OR 0.11, 95% CI 0.01 to 0.91, *P* = 0.04). Forest plots for the postoperative and final follow-up dysphagia evaluations are presented in Fig. 8.

### Subsidence

**Fig. 9:**
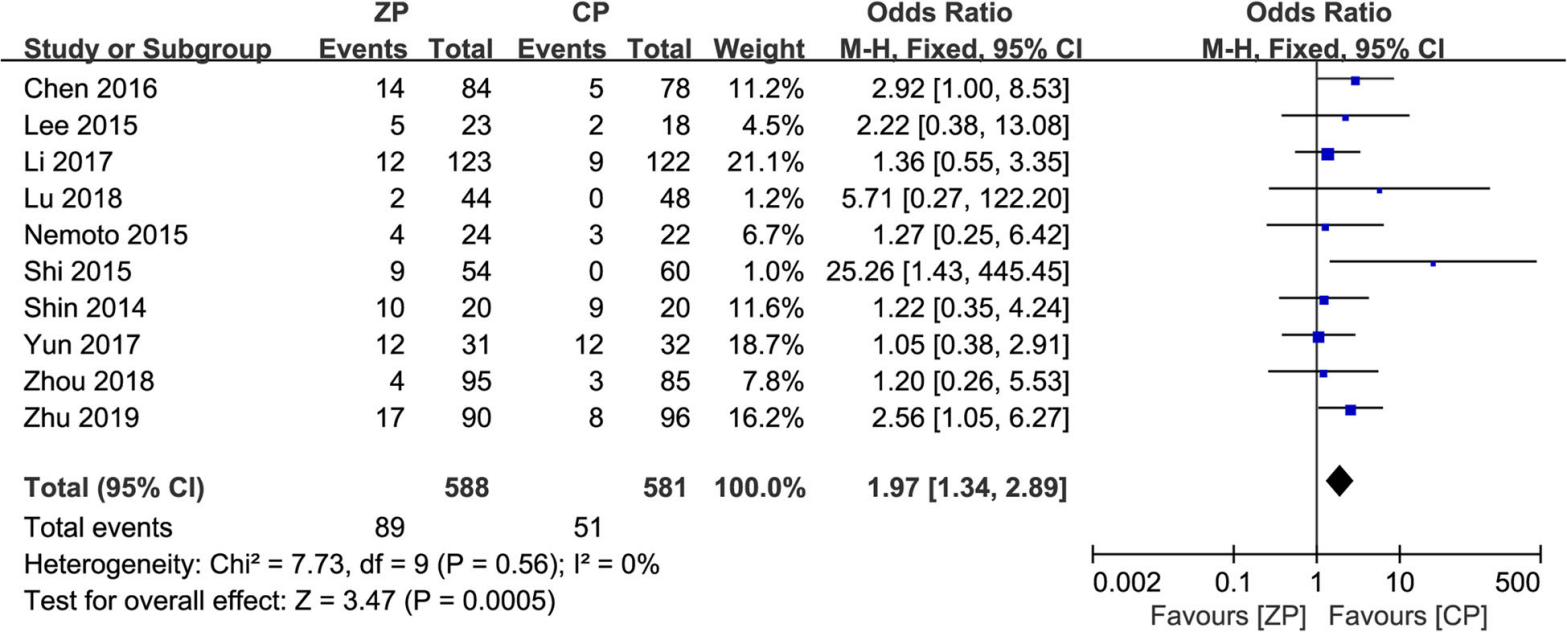
The forest plot shows subsidence of anterior cervical discectomy and fusion by using zero-profile anchored cage versus cage-plate construct at the final follow-up

**Fig. 10:**
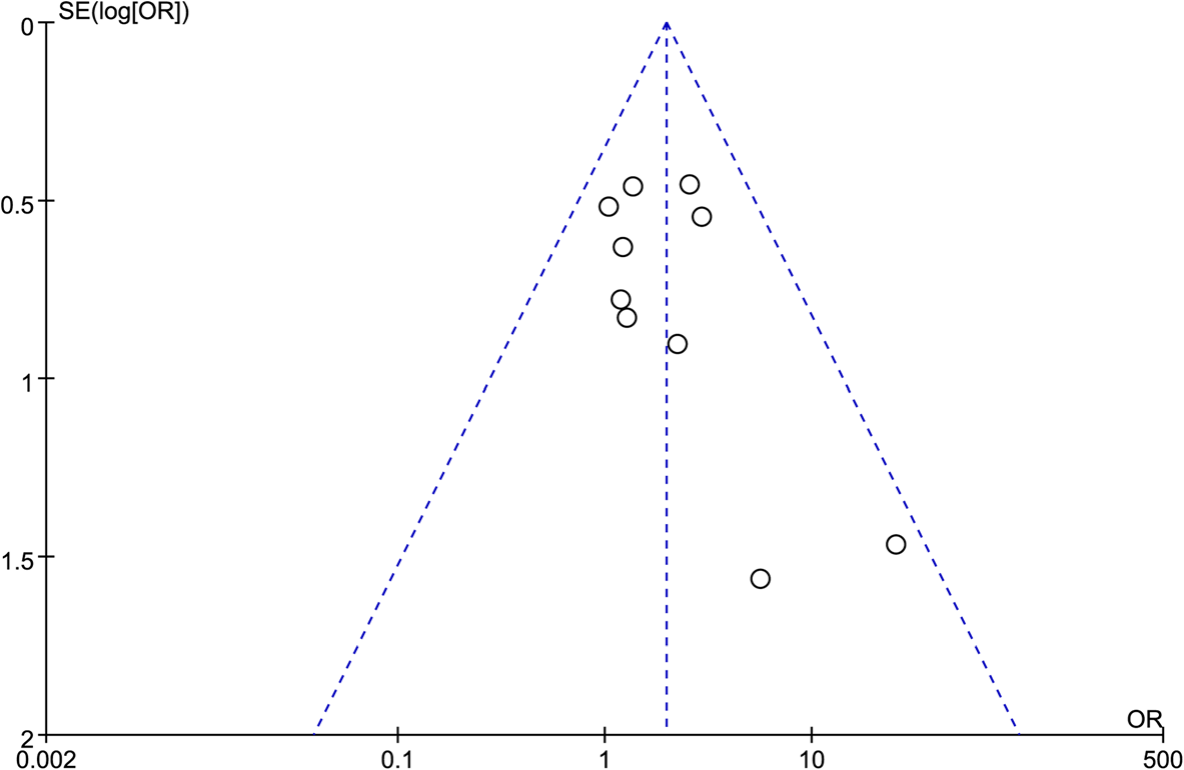
A funnel plot for publication bias of subsidence at the final follow-up

A total of ten studies were included in the comparison of the incidence of subsidence between the ZP and CP groups [2, 9–13, 23–26]. The subsidence rates were 15.1% (89/588) in the ZP group and 8.8% (51/581) in the CP group. The subsidence rate was significantly higher in the ZP group patients (OR 1.97, 95% CI 1.34 to 2.89, *P* = 0.0005). The forest plot analysis of subsidence and the funnel plot evaluation of publication bias are presented in Figs. 9 and 10, respectively.

We performed a subgroup analysis stratified by the definition of subsidence, which included subsidence ≥ 2 mm in five studies [2, 9, 12, 13, 25] and ≥ 3 mm in four studies [10, 11, 23, 24]. In the ZP group, the incidence was 16.5% (42/255) and 12.3% (30/243) with the definition of ≥ 2 mm and ≥ 3 mm, respectively. Correspondingly, it was 10.2% (26/254) and 7.4% (17/231) in the CP group. These results showed that there was a higher risk of subsidence in the ZP group, regardless of whether the definition of subsidence was ≥ 2 mm (OR 1.78, 95% CI 1.03 to 3.06, *P* = 0.04) and ≥ 3 mm (OR 1.98, 95% CI 1.00 to 3.91, *P* = 0.05).

**Fig. 11:**
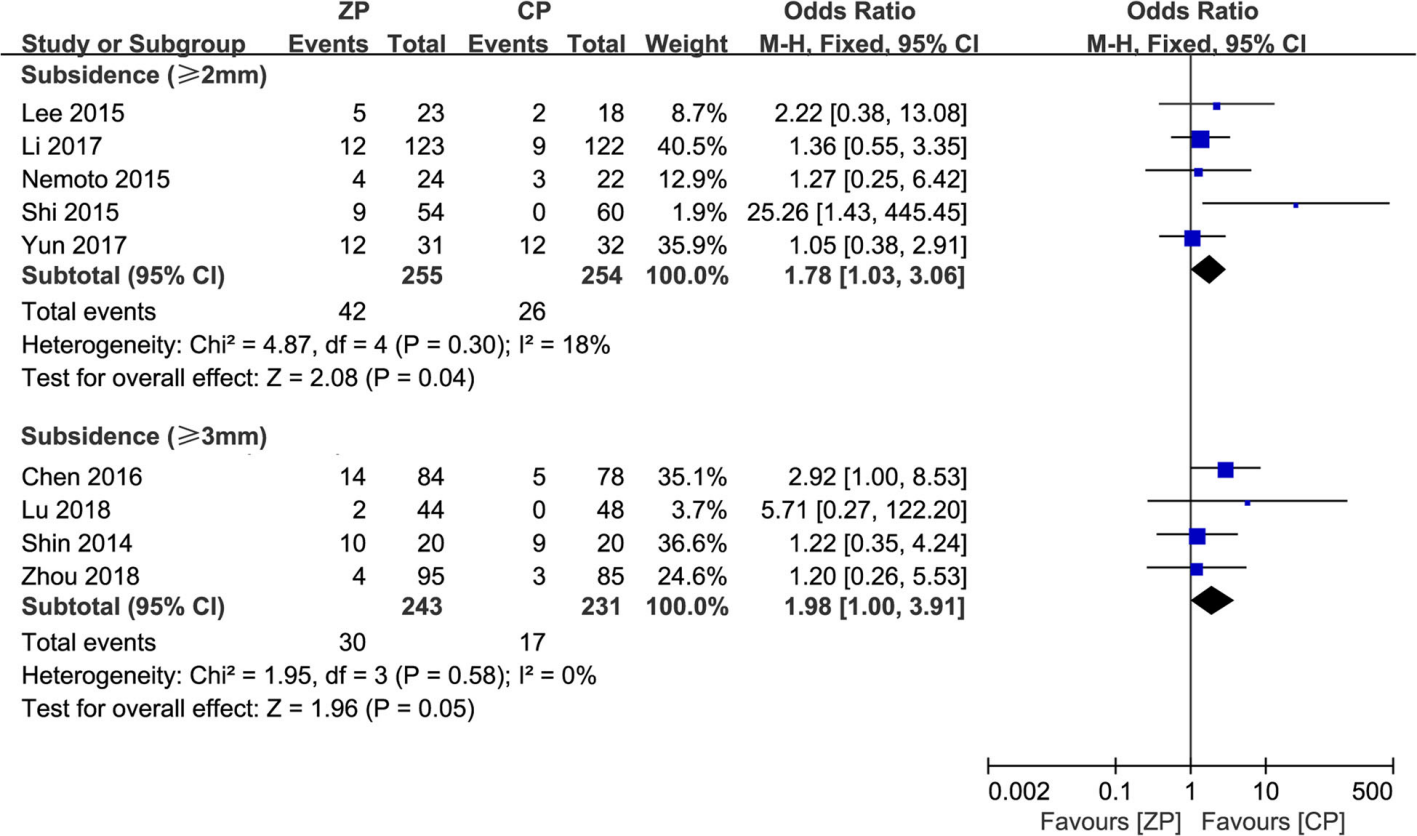
The forest plot shows subgroup analysis for subsidence stratified by definition of ≥ 2 mm and ≥ 3 mm after anterior cervical discectomy and fusion with zero-profile anchored cage compared to cage-plate construct

**Fig. 12:**
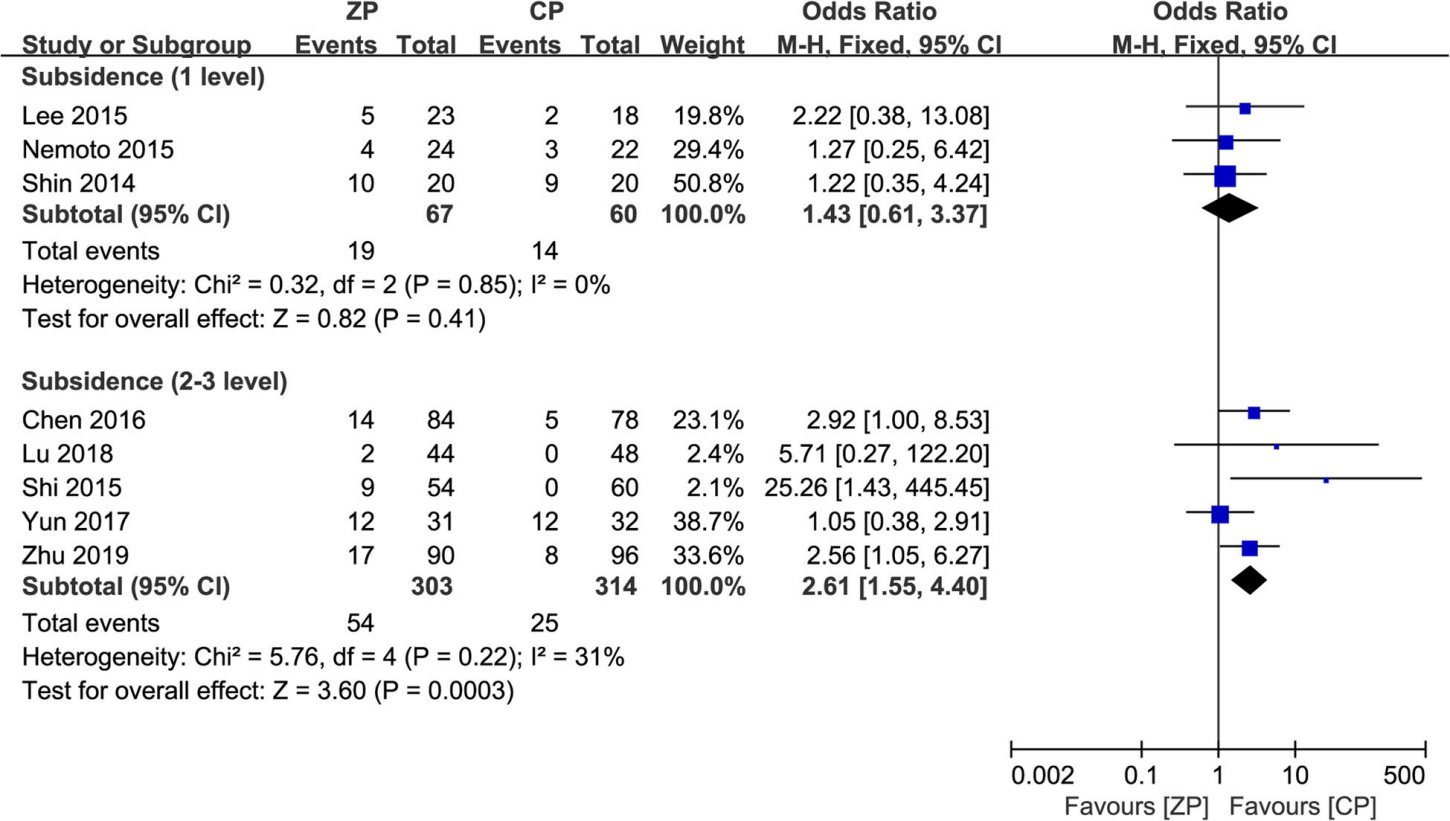
The forest plot shows subgroup analysis for subsidence stratified by one-level and multilevel surgery after anterior cervical discectomy and fusion with zero-profile anchored cage compared to cage-plate construct

A subgroup analysis stratified by the quantity of operative segments was also performed, which included single-level surgery in three studies [2, 12, 24] and multilevel surgery in five studies [9, 11, 23, 25, 26]. In the single-segment operations, the incidence of subsidence between the ZP and CP groups was not significant (OR 1.43, 95% CI 0.61 to 3.37, *P* = 0.41). For the multilevel surgeries, the ZP group had a higher incidence of subsidence (OR 2.61, 95% CI 1.55 to 4.40, *P* = 0.0003). The forest plots for the subgroup analysis of subsidence are described in Figs. 11 and 12, respectively.

## Discussion

ACDF is a classic procedure in treating cervical degenerative diseases that can remove the prominent disk and osteophyte and relieve compressions of the spinal cord and nerve root. It is effective and widely used in the treatment of patients with CDDD [1]. The anterior plating system not only stabilizes the upper and lower vertebral bodies, but also improves the rate of intervertebral fusion and avoids the risk of pseudarthrosis [3, 4]. Furthermore, it can maintain the cervical sagittal alignment and prevent graft extrusion or subsidence [2].

However, the application of the anterior plate has a high incidence of postoperative dysphagia [5–8]. In response to this problem, various stand-alone anchored spacers have been designed for clinical treatment. Wang et al. [8] found that the dysphagia rate was only 4.5% after one-level ACDF with a Zero-P device, which was significantly lower than 32% dysphagia rate reported when using the cage-plate construct. Yang et al. [5] reported that the Zero-P device has a lower incidence of dysphagia than the anterior plating system after multilevel fusion (4.3% versus 25%, *P* = 0.04). Hofstetter et al. [7] also found that the use of zero-profile anchored spacers (MC+ or ROI-C) had significantly lower rates of dysphagia than conventional plate instruments after surgery (2.9% versus 20%, *P* = 0.027).

Subsidence is another equally important adverse event after ACDF. It usually refers to an object with a greater elastic modulus (e.g., cage or spacer) entering another object of relatively lower elastic modulus (e.g., vertebral body). The subsidence of the cage causes the loss of intervertebral disk height, which can result in narrowing of the foramen, nerve root compression, and pseudoarthrosis due to cervical instability [27]. Eventually, the cervical spine loses physiological curvature, resulting in kyphosis. This means that segmental subsidence arouses significant morbidity during the postoperative period [18].

A systematic review of seventy-one studies reported that the mean incidence of cage subsidence after ACDF was 21% (ranging from 0 to 83%) [27]. Many studies have reported that the use of plates can reduce the incidence of subsidence after ACDF. Lee et al. [18] confirmed that the nonuse of plates was a risk factor for subsidence and had a significantly high subsidence rate after single-level ACDF (40% versus 12.5%, *P* = 0.025). Kim et al. [28] found that the postoperative subsidence rate of patients using a stand-alone cage was higher than that of patients using a cage-plate construct after two-level fusion (66.6% versus 30%, *P* = 0.049), which indicated that the use of a plate could maintain the height of the intervertebral disk and play a pivotal role in reducing postoperative subsidence. However, the incidence of subsidence for ZP compared to CP after reviewing the literature is not clear. Therefore, we conducted this meta-analysis to determine whether there is a statistically high risk of cage subsidence after ACDF with ZP.

Ten trials including 10 cohorts with a total of 626 patients were included in this study. There were no significant differences in blood loss, JOA score, NDI score, fusion rate, or cervical alignment at the final follow-up between the two devices by meta-analysis. Additionally, we found that the incidence of dysphagia in the CP group was significantly higher than that in the ZP group at each follow-up time, which was consistent with previous studies. Xiao et al. [29] conducted a meta-analysis that included 1066 patients to compare the postoperative dysphagia rate between zero-profile anchored spacers and cage-plate constructs after ACDF. Their study found that the ZP group had a significantly lower risk of dysphagia. Nambiar et al. [30] and Tong et al. [31] also found that the ZP group achieved a lower incidence of dysphagia after single-level and multilevel fusion, respectively.

The results of this study suggested that the ZP group was associated with a high incidence of subsidence, when compared with the CP group. Considering the different definitions of subsidence used in the above ten studies, we conducted subgroup analysis of subsidence defined as ≥ 2 mm and subsidence defined as ≥ 3 mm. However, we found that the incidence of subsidence in the ZP group was still higher than that in the CP group, regardless of definition. Therefore, it could be considered that different definitions of subsidence did not affect the consequences. Then, we performed subgroup analysis according to the quantity of operative segments. There was no significant difference in the rate of subsidence between the ZP group and CP group after single-segment ACDF, while the subsidence rate of the ZP group was significantly higher than that of the CP group after multisegment ACDF. Therefore, we believed that there was no significant difference in the occurrence of cage subsidence after single-level ACDF with ZP or CP. However, considering plate-related complications and the incidence of dysphagia after surgery, we preferred ZP for the treatment of single-level CDDD. For multilevel surgery, due to the high subsidence rate of ZP and with the goal of avoiding subsequent loss of cervical alignment and kyphosis deformity, CP should be preferred without considering the risk of dysphagia.

Our meta-analysis was restricted by some limitations. First, retrospective and nonrandom studies were included in this study, which inevitably gave rise to selection bias. Second, only 10 studies were included, the sample sizes were relatively small, and the follow-up times differed. In addition, a variety of zero-profile devices were used, which could affect the accuracy of our conclusions, although the devices had similar fixation mechanisms and structures. Finally, clinical heterogeneity may have resulted from the various surgical approaches and types of implants used in different research centers.

To the best of our knowledge, this study was the first meta-analysis to compare the subsidence of ZP to that of CP after ACDF, despite the above shortcomings. Our study showed that ZP had a higher postoperative subsidence than CP, especially in multilevel surgery. Moreover, both devices were safe and effective and achieved similar clinical outcomes. A high-quality, large-sample RCT is required to validate our findings in the future.

Currently, there is no consensus on the relationship between subsidence and clinical effectiveness. The research performed by Lee et al. [18] found that cage subsidence was associated with higher neck and arm VAS scores. Kim et al. [20] also reported that the existence of subsidence was significantly related to unfavorable clinical outcomes at all follow-up assessments, which were evaluated by Odom’s criteria. However, Park et al. [32] recruited and divided 77 patients into subsidence and non-subsidence groups, and found that subsidence did not correlate with fusion rate or clinical outcomes. Yson et al. [33] also demonstrated that subsidence did not seem to be predictive of clinical outcomes of ACDF. More longitudinal multicenter RCTs should explore the role of subsidence in clinical prognosis. Furthermore, the surgeon should pay more attention to the deuteropathy of subsidence. We suggest that it is more favorable to use zero-profile implants for one-level surgery and plate fixation in multilevel fusion.

## Conclusion

The ZP, which has been used routinely in recent years, may not reduce the subsidence rate compared to CP. There was no significant difference in subsidence between these two fixed apparatuses after single-level ACDF, while the risk of subsidence in ZP was significantly higher after multilevel fusion. Although there was a high incidence of swallowing discomfort, we would suggest that the anterior plate should be used in multilevel surgery, if possible to reduce subsidence and adverse clinical symptoms in the long term. We conclude that this study provides new opinions for the rational use of ZP.

## Data Availability

Not applicable.

## Abbreviations

ZP: Zero-profile anchored cage
CP: Cage-plate construct
ACDF: Anterior cervical discectomy and fusion
OR: Odds ratio
CI: Confidence interval
CDDD: Cervical degenerative disk disease
PRISMA: Preferred Reporting Items for Systematic Reviews and Meta-Analysis
JOA: Japanese Orthopaedic Association
NDI: Neck Disability Index
WMD: Weighted mean difference
